# A new species of *Paraonis* and an annotated checklist of polychaetes from mangroves of the Brazilian Amazon Coast (Annelida, Paraonidae)

**DOI:** 10.3897/zookeys.740.14640

**Published:** 2018-02-27

**Authors:** Rannyele Passos Ribeiro, Paulo Ricardo Alves, Zafira da Silva de Almeida, Christine Ruta

**Affiliations:** 1 Universidad Autónoma de Madrid, Cantoblanco, 28049, Madrid, Spain; 2 Universidade Federal Fluminense, Programa de Pós-Graduação em Biologia Marinha e Ambientes Costeiros, Departamento de Biologia Marinha. Laboratório de Sistemática e Ecologia de Polychaeta, Niterói, 24020-141, Rio de Janeiro, Brazil; 3 Universidade Estadual do Maranhão, Laboratório de Pesca e Ecologia Aquática, São Luís, 65055-000, Maranhão, Brazil; 4 Universidade Federal do Rio de Janeiro, Campus UFRJ-Macaé Professor Aloísio Teixeira. Núcleo em Ecologia e Desenvolvimentto Sócio-Ambiental de Macaé, Grupo de Sistemática e Ecologia de Organismos Bentônicos. Macaé, 27965-045, Rio de Janeiro, Brazil

**Keywords:** annelid, mangrove, Maranhão, new records, *Paraonis*, taxonomy

## Abstract

The polychaete fauna from the mangroves of the Amazon Coast in Maranhão state, Brazil, is reported in this study. Fourteen species are listed, namely *Alitta succinea* (Leuckart, 1847); Arabella (Arabella) iricolor Montagu, 1804; *Capitella capitata* (Fabricius, 1780) complex; Exogone (Exogone) breviantennata Hartmann-Schröder, 1959; *Heteromastus filiformis* (Claparède, 1864); *Isolda pulchella* Müller, 1858; *Mediomastus californiensis* Hartman, 1944; *Namalycastis fauveli* Nageswara Rao, 1981; *Namalycastis geayi* (Gravier, 1901); *Namalycastis senegalensis* (Saint-Joseph, 1901); *Nephtys simoni* Perkins, 1980; *Paraonis amazonica* **sp. n.**; *Sigambra bassi* (Hartman, 1945); and *Sigambra grubii* Müller, 1858. Among them, *Namalycastis fauveli* and *Namalycastis geayi* are recorded for the first time in Brazil. *Paraonis amazonica* **sp. n.** is a new species for science, characterized by a rounded prostomium, 4–8 pairs of foliaceous branchiae, absent eyes, and two types of modified neurochaetae, acicular and hook-shaped.

## Introduction

The two largest rivers that drain South America, the Amazon and the Orinoco, are respectively, the first and third largest rivers in the world in terms of water volume (Degens et al. 1991). The Orinoco and Amazon Rivers are responsible for the discharge of an enormous amount of freshwater and sediment into the ocean, representing nearly 20 % of the total global annual freshwater (Hu et al. 2004; Miloslavich et al. 2011). These rivers have been recognized as zoogeographic barriers to the dispersal of marine fauna between the Caribbean and southwestern Atlantic (Gilbert 1972; Floeter and Gasparini 2000). Accordingly, they influence the Brazilian Northern Coast, also known as the Brazilian Amazon Coast, which extends from the north of the Amapá State to the Gulf of Maranhão and represents 35 % of the entire Brazilian Coast (Silveira 1964; Sousa et al. 2008). This region is characterized by a variety of poorly known estuarine and marine ecosystems with very diverse habitats (Couto et al. 2003).

Most of what is known about the marine biodiversity of the Brazilian Amazon Coast is related to fishing and mangrove habitats Nevertheless, the REVIZEE Program – Living Resources in the Exclusive Economic Zone provided important information regarding the continental shelf and offshore area (Miloslavich et al. 2011). In general, macrobenthos assemblages are one of the least known of Brazil (Amaral and Jablonski 2005; Miloslavich et al. 2011). The lack of studies in this region represents one of the major gaps in the knowledge of the biodiversity of Brazilian polychaetes (see Lana et al. 2009). The main studies concerning polychaetes in this region include ecological research with records in the coast of the states of Maranhão (see Ribeiro and Almeida 2014) and Pará (Rosa-Filho et al. 2006; Morais and Lee 2014) recording a total of 27 species and 24 families.

In this study, polychaetes collected in the mangrove of São Marcos Bay in the Gulf of Maranhão were examined. The current study contributes to increasing the knowledge of polychaetes in the South Atlantic, particularly in the Amazon coastal zone. This is the first taxonomic study with a focus on the polychaete fauna from Maranhão and includes new records and the description of a new species.

## Materials and methods

**Figure 1.**
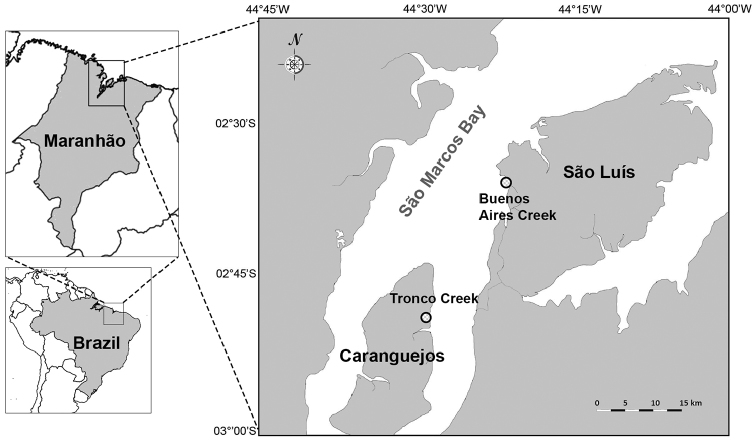
Study area. Buenos Aires and Tronco creeks in Maranhão, Brazilian Amazon Coast.

Mangrove specimens from the Gulf of Maranhão were collected between April of 2010 and June of 2012 from two creeks in São Marcos Bay: Buenos Aires at the São Luís (02°35'56"S, 44°21'11.8"W) and Tronco at the Caranguejos Island (02°49'33.6"S, 44°28'51.1"W) (Fig. 1). Along of a 100 m transect, nine sediment samples were taken using a corer (20 cm long and 10 cm diameter) at the intertidal region of each creek. Samples were washed through a 0.5 mm mesh sieve with filtered freshwater in the laboratory; specimens were fixed in 4 % formaldehyde and then transferred to 70 % ethanol for long-term storage.

Polychaetes were identified at the species level using stereo (Olympus SZX-16) and light microscope (Olympus CX31). Specimens were prepared for scanning electron microscopy (SEM) by critical point drying, prior to being mounted on stubs and coated with gold (200 A thick). These specimens were observed and analyzed in the Jeol JSM-6390LV scanning electron microscope of the Museu Nacional/Universidade Federal do Rio de Janeiro (MNRJ). Light microscopy images were taken with a camera attached to a Leica M205A stereo microscope and a Zeiss Axio Scope microscope. Drawings and pictures were processed using Adobe (San Jose, CA, USA) Photoshop CS6.

The specimens and type material referent to the new species were deposited in the research collection Coleção Científica de Invertebrados Marinhos e Costeiros (NPM) of the Núcleo em Ecologia e Desenvolvimento Sócio-Ambiental de Macaé (NUPEM), Macaé, Brazil, and in the Museo Nacional de Ciencias Naturales (MNCN), Madrid, Spain. Additionally, we elaborated a list of some Brazilian records of the species identified here, taking into account those reported by studies formally published (Suppl. material 1). We designated up to one previous record for each Brazilian state indicated in the species distribution section, plus original description, when applicable. Our criteria to select the studies were, preferably: taxonomic approach, ecological approach providing voucher-specimens, ecological approach without voucher-specimens.

Other abbreviations cited in this study:

**BMHN** British Museum (Natural History), London.

**IBUFRJ** Coleção de Polychaeta do Museu Nacional, Rio de Janeiro.

**MCEM-BPO** Centro de Estudos do Mar, Universidade Federal do Paraná, Pontal do Paraná.

**MZUSP** Museo Nacional de Ciencias Naturales.

**MPEG.ANL** Museu Paraense Emílio Goeldi.

**POLY-UFPB** Coleção de Invertebrados Marinhos Paulo Young, Universidade Federal da Paraíba, Paraíba.

**UK** United Kingdom.

**USA** United States of America.

**USNM** United States National Museum, Smithsonian Institution, Washington.

**ZMH** Zoologisches Staatsinstitut und Zoologisches Museum Hamburg, Hamburg.

**ZUEC** Coleção de Polychaeta do Museu de Zoologia “Prof. Adão José Cardoso”, Universidade Estadual de Campinas, Campinas.

## Taxonomy

A total of eight families, eleven genera, and 14 species were identified; new records of *Namalycastis geayi* and *Namalycastis senegalensis* (Nereididae) and a new species of *Paraonis* (Paraonidae) are reported from Brazil.

### Phylum Annelida

#### Subclass Errantia

##### Order Eunicida

###### Family Oenonidae Kinberg, 1865

####### Genus *Arabella* Grube, 1850

######## Arabella (Arabella) iricolor

Taxon classificationAnimaliaEunicidaOenonidae

(Montagu, 1804)

Fig. 2

######### Type locality.

Devonshire, England, United Kingdom (50°34'N, 3°34'W; estimated geolocation).

######### Material examined.

São Luís, 02°35'56"S, 44°211'1.8"W: two specimens, 18 March 2012 (NPM-Pol 115); one specimen, 1 June 2012 (NPM-Pol 090); one specimen, 18 November 2011 (NPM-Pol 886); complete and incomplete specimens.

######### Distribution.

Pacific Ocean: New Zealand, Philippines, USA, Peru. Indian Ocean: Red Sea. Atlantic Ocean: Ireland, UK, France, Mediterranean Sea, Marmara Sea (Turkey), Mauritania, South Africa, USA, Mexico, Caribbean Sea, Brazil (states of Maranhão, Bahia, São Paulo and Paraná, see Suppl. material 1).

######### Remarks.

**Figure 2.**
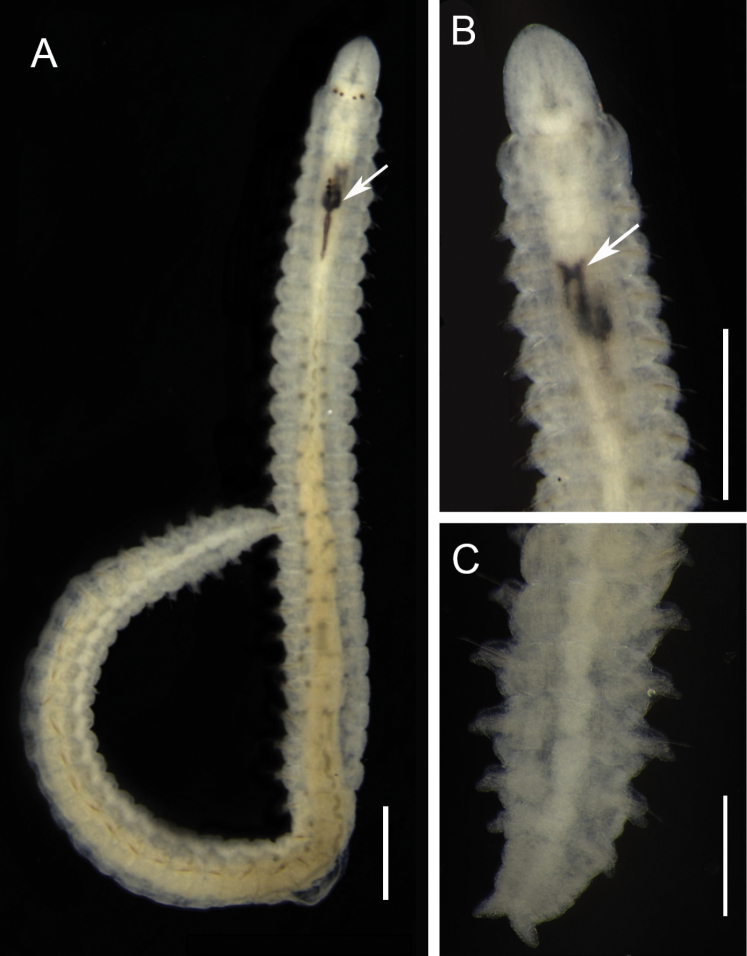
Arabella (Arabella) iricolor, fixed specimen. **A** Whole body, dorsal view, arrow point to maxillae and carriers **B** Anterior end, ventral view, arrow point to mandibles **C** Posterior end, dorsal view. Scale bars: 0.5 mm (**A, B**), 0.3 mm (**C**).

Arabella (Arabella) iricolor was described to the south coast of Devonshire (UK) as *Nereis iricolor* (Montagu, 1804). The description of specimens from the Caribbean Sea (Augener 1927) closely reSEMbles specimens in this study, which were identified as this species due to the characteristics: ventralmost chaeta tapering gradually to guards in median and posterior chaetigers, the absence of hooded acicular chaetae, maxilla MxI unidentate and posterior post-chaetal lobe shorter than chaetae. Body surface whitish was observed in small fixed individuals, probably juveniles (Fig. 2A–C). (Montagu 1804). The species was recorded in ecological studies of the continental shelf, intertidal zone, coral reefs, estuaries, and mangroves (Paiva 1993, Santa-Isabel et al. 2000), but apparently, the material was not deposited in any collection and was not available for comparison. Previous record from Maranhão reports specimens found in mangroves (Oliveira and Mochel 1999). This species has been described with worldwide distribution and is probably a complex of species (Colbath 1989; Zanol and Ruta 2015). Studies on the variation of the symmetry in maxillae and modified ventral chaetae should be conducted to know the polymorphism in species of the genus *Arabella* (Steiner and Amaral 2009). That would be a challenge to species identification and new descriptions, once Oenonidae species are usually collected in low densities (Zanol 2010, Zanol and Ruta 2015).

##### Order Phyllodocida

###### Family Nephtyidae Grube, 1850

####### Genus *Nephtys* Cuvier, 1817

######## Nephtys simoni

Taxon classificationAnimaliaPhyllodocidaNephtyidae

Perkins, 1980

Fig. 3

######### Type locality.

Hutchinson Island, Florida, USA (27°21.6'N, 80°13.2'W; original geolocation).

######### Material examined.

São Luís, 02°35'56"S, 44°21'11.8"W: one specimen, 23 April 2010 (NPM-Pol 868); four specimens, 21 October 2010 (NPM-Pol 869); one specimen, 27 January 2011 (NPM-Pol 870); four specimens, 27 January 2011 (NPM-Pol 871); one specimen, 6 September 2011 (NPM-Pol 872); one specimen, 18 December 2011 (NPM-Pol 873); complete and incomplete specimens. Caranguejos Island, 02°49'33.6"S, 44°28'51.1"W: 31 specimens, 20 October 2010 (NPM-Pol 874); 12 specimens, 17 March 2012 (NPM-Pol 455); six specimens, 5 September 2011 (NPM-Pol 875); complete and incomplete specimens.

**Figure 3.**
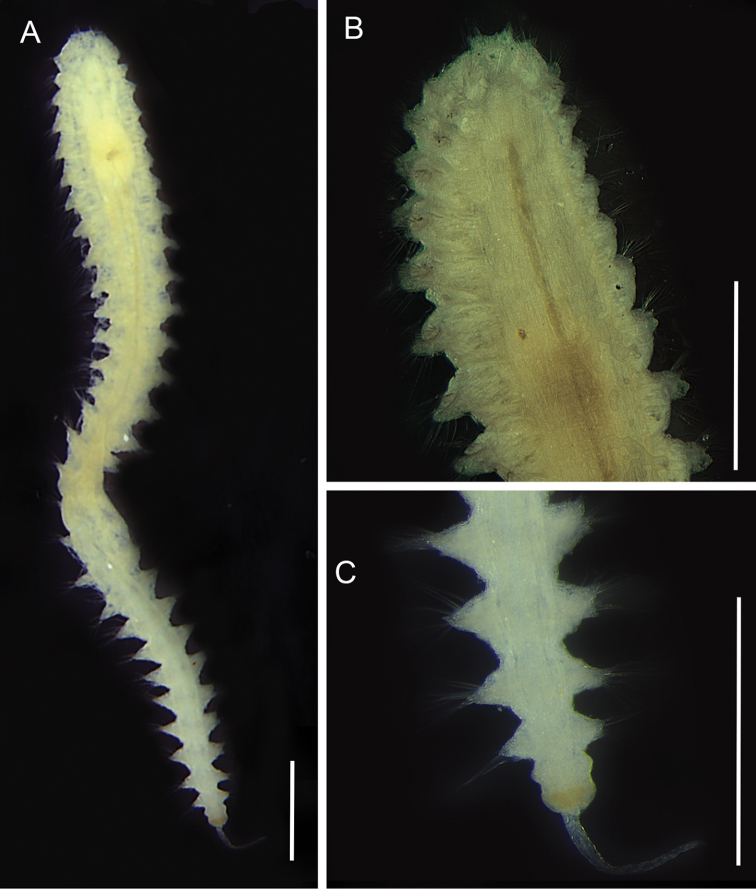
*Nephtys simoni*, fixed specimen. **A** Whole body, dorsal view **B** Anterior end, dorsal view **C** Posterior end, dorsal view. Scale bars: 0.5 mm.

######### Distribution.

Atlantic Ocean: Mediterranean Sea, USA, Mexico, Brazil (states of Pará, Maranhão, São Paulo, see Suppl. material 1).

######### Remarks.

First record for Maranhão. The specimens present the characters that define *Nephtys simoni* Perkis, 1980, such as interramal branchiae from the third chaetiger; proboscis with long middorsal and midventral subdistal papilla and 23 conical papilla distal, dorsal cirrus linked to pre-chaetal cirrus, short and finger-like lobes. Individuals that have one pair of eyespots and median reddish pigmentation in the prostomium were reported for juveniles by Perkins (1980). In this study, some specimens presented eyespots, but not the reddish pigmentation pattern. Specimens of *N. simoni* have been reported in estuarine areas as in the type locality (Perkins 1980) and in Amazon mangroves (Silva et al. 2011). In Brazil, the specimens recorded as *Nephtys simoni* in Paranaguá Bay, Paraná (Lana 1986) are in fact *Nephtys californiensis* Hartman, 1938 (Rizzo and Amaral 2007).

###### Family Nereididae Blainville, 1818

####### Genus *Alitta* Kinberg, 1865

######## Alitta succinea

Taxon classificationAnimaliaPhyllodocidaNephtyidae

(Leuckart, 1847)

Fig. 4

######### Type locality.

Helgoland and Cuxhaven, Germany (53°53'N, 8°37'E; estimated geolocation).

######### Material examined.

São Luís, 02°35'56"S, 44°21'11.8"W: one specimen, 6 September 2011 (NPM-Pol 083); two specimens, 27 January 2011 (NPM-Pol 876); complete and incomplete specimens. Caranguejos Island, 02°49'33.6"S, 44°28'51.1"W: one specimen, 20 October 2010 (NPM-Pol 877); complete and incomplete specimens.

**Figure 4.**
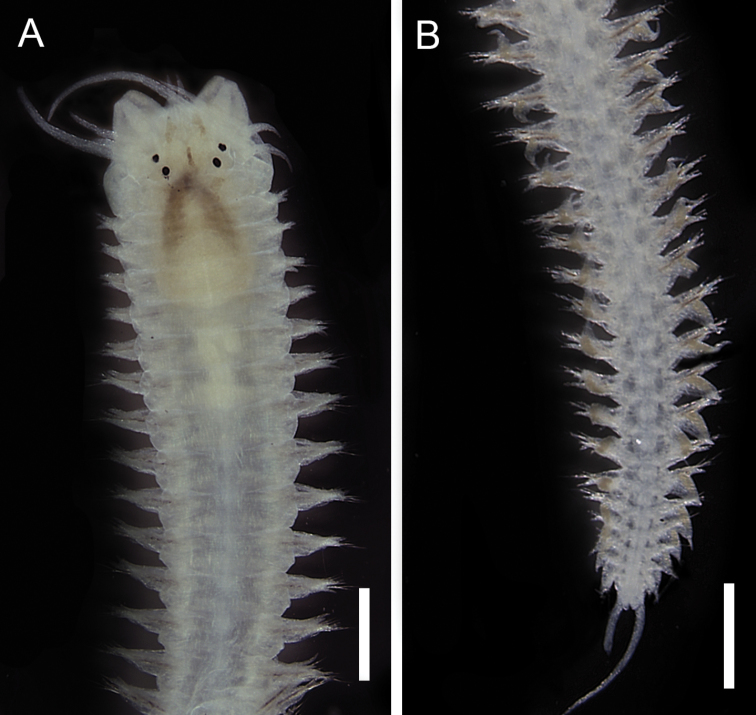
*Alitta succinea*, fixed specimen. **A** Anterior end, dorsal view **B** Posterior end, dorsal view. Scale bars: 0.5 mm.

######### Distribution.

Pacific Ocean: Australia, New Zealand, USA, Mexico. Indian Ocean: Red Sea. Atlantic Ocean: North Sea, Mediterranean Sea, South Africa, Canada, USA, Caribbean Sea, Brazil (state of Pará, Maranhão, Pernambuco, Bahia, Espírito Santo, Rio de Janeiro, São Paulo, Paraná, Santa Catarina and Rio Grande do Sul, see Suppl. material 1).

######### Remarks.

This species was described as *Nereis succinea* (Leuckart 1847), transferred to genus *Neanthes* (Imajima 1972), and later to *Alitta* (Bakken and Wilson 2005). The specimens examined in this study share the features of the specimens re-described by Villalobos-Guerrero and Carrera-Parra (2015), such as paragnaths present in all areas of the pharynx, homogomph spinigerous notochaetae, neurochaetae with heterogomph spinigers and homogomph and heterogomph falcigers and the widely expanded notopodial ligule in posterior parapodia. However they are smaller (major individual measuring 3.5 mm of length from the prostomium to the 25^th^ chaetiger) than those described from the Caribbean Sea (Espinosa et al. 2007) and southern-southeastern Brazil (Amaral et al. 2005). On the other hand, specimens from northeastern Brazil measuring less than 5 mm length from the prostomium to the 25^th^ chaetiger are considered recruits (Sette et al. 2013). Therefore, we suggest all the individuals collected in this study are juveniles. *Alitta succinea* is widely distributed in the world with records in different environments. This species was recorded in mangroves from the Caribbean Sea (Londoño-Mesa et al. 2002) and Brazil, including a record in Maranhão state (Mochel 1997). This species is reported in environments with different salinity levels and has been considered as a euryhaline species (Sato 2013).

####### Genus *Namalycastis* Hartman, 1959

######## Namalycastis fauveli

Taxon classificationAnimaliaPhyllodocidaNephtyidae

Nageswara Rao, 1981

Fig. 5

######### Type locality.

Estuary of Tachin River, Thailand (13°44'N, 100°30'E; original geolocation).

######### Material examined.

São Luís, 02°35'56"S, 44°21'11.8"W: one specimen, 6 September 2011 (NPM-Pol 883). Caranguejos Island, 02°49'33.6"S, 44°28'51.1"W: one specimen, 26 January 2011 (NPM-Pol 086); three specimens, 28 March 2011 (NPM-Pol 109); one specimen, 22 April 2010 (NPM-Pol 878); three specimens, 17 August 2010 (NPM-Pol 879); two specimens, 2 June 2012 (NPM-Pol 880); four specimens, 5 September 2011 (NPM-Pol 881). Complete and incomplete specimens.

######### Distribution.

Indian Ocean: Thailand, India. Atlantic Ocean: Brazil (Maranhão state).

######### Diagnosis.

Body widest mid-anteriorly. Prostomium anteriorly shallowly cleft or cleft absent. Antennae minute. Notochaeta present. Heterogomph setae with boss extremely prolonged. Supra-neuroacicular falcigers in chaetiger 10 with blades slightly curved (Glasby 1999).

######### Description.

**Figure 5.**
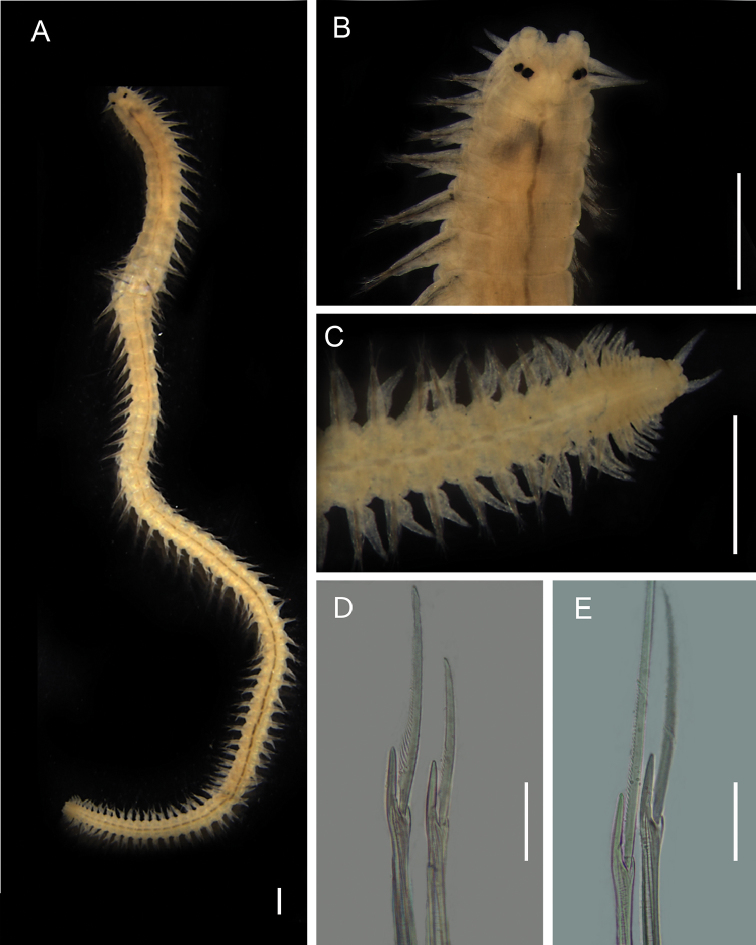
*Namalycastis fauveli*, fixed specimen, NPM-Pol 883. **A** Whole body, dorsal view **B** Anterior end, dorsal view **C** Posterior end, ventral view **D** Neurochaetae supra-acicular, 10^th^ chaetiger **E** Neurochaeta spiniger supra-acicular, 10^th^ chaetiger. Scale bars: 0.5 mm (**A–C**), 0.01 mm (**D, E**).

Based on specimens NPM-Pol 878 and 883. Complete specimen with 17.3 mm long, 0.72 mm wide (chaetiger 10), and 79 chaetigers (Fig. 5A). Body widest mid-anteriorly, gradually tapering anteriorly and posteriorly. Dorsum convex. Epidermal pigment absent. Prostomium trapezoidal, some individuals with lateral indentation on prostomium. Prostomium anterior end smooth or with a shalow cleft (Fig. 5B). Narrow longitudinal groove extending form tip to mid-posterior prostomium. Antennae short, extending short of palpophore anterior end, laterally inserted. Two pair of eyes transversally arranged on prostomium. Four pairs of tentacular cirri with indistinct cirrophores and smooth cirrostyles. Posterodorsal pair extending posteriorly to third chaetiger. Pharynx smooth, lacking paragnaths or papillae. Parapodia sesquirramous (sub-birramous). Dorsal cirri increasing in length posteriorly. Neuropodial acicular ligulae bilobed. Notochaeta as sesquigomph spinigers present from third chaetiger. Supra-acicular neurochaeta as sesquigomph spinigers on postacicular fascicle and heterogomph falcigers on preacicular fascicle (Fig. 5D, E). Sub-acicular neurochaetae as heterogomph spinigers on postacicular fascicle and heterogomph falcigers in preacicular fascicle. Supra-acicular sesquigomph spinigers shaft with boss 1.2×–1.5× length of collar. Shaft of heterogomph chaeta with boss prolonged. Supra-acicular falcigers in chaetiger 10 with blades slightly curved, blades length 8.0×–9.5× width of shaft head. Sub-acicular falcigers blades in chaetiger 10 length, 8.2×–11.4× (dorsal-most) and 6.0×–7.3× (ventral most) width of shaft head. Sub-acicular spinigers in anterior region of body with blades finely serrated. Chaeta pale. Aciculae dark brown. Pigidium buttom-shaped (Fig. 5C). Anus terminal. Anal cirri smooth and subconical, arising ventro-laterally.

######### Colour.

Specimens in alcohol yellow. No pigment visible throughout the body.

######### Remarks.

First species record for the America. These specimens present some differences from the original description (Nageswara Rao 1981), such as a dorsal surface convex, body less arched mid-anteriorly, longer antennae and tentacular cirri (Fig. 2A). However, the projection of heterogomph chaetae with an extremely long boss supports this identification for this species (Fig. 2B, C). The differences found are probably because the specimens in this study are juveniles by the smaller size (around 15 mm long, and 80 chaetigers), compared to type material, 21–45 mm long, 134–282 chaetigers, after Glasby (1999). Some Namanereidinae species, as *Namalycastis abiuma*, can have juveniles with blades longer and up to 80 chaetigers. *Namalycastis fauveli* is recorded in estuarine beaches and coastal lagoons in the type locality (Nageswara Rao 1981) and in mangroves of this study.

######## Namalycastis geayi

Taxon classificationAnimaliaPhyllodocidaNephtyidae

(Gravier, 1901)

Fig. 6

######### Type locality.

Ouanary, French Guiana (4°12'N, 51°39'W; estimated geolocation).

######### Material examined.

Caranguejos Island, 02°49'33.6"S, 44°28'51.1"W: one specimen, 17 December 2011 (NPM-Pol 082); one specimen, 27 March 2011 (NPM-Pol 882); one specimen, 20 October 2010 (NPM-Pol 884); all incomplete specimens.

######### Distribution.

Atlantic Ocean: French Guiana, Brazil (state of Maranhão).

######### Diagnosis.

Prostomium anterior end smooth or with a shallow cleft. Antennae extending short of the palpophore tip or of the prostomium tip. Two pairs of eyes nearly longitudinally arranged. Dorsal cirri short, similar in length throughout the body. Notochaetae present (Glasby 1999).

######### Description.

**Figure 6.**
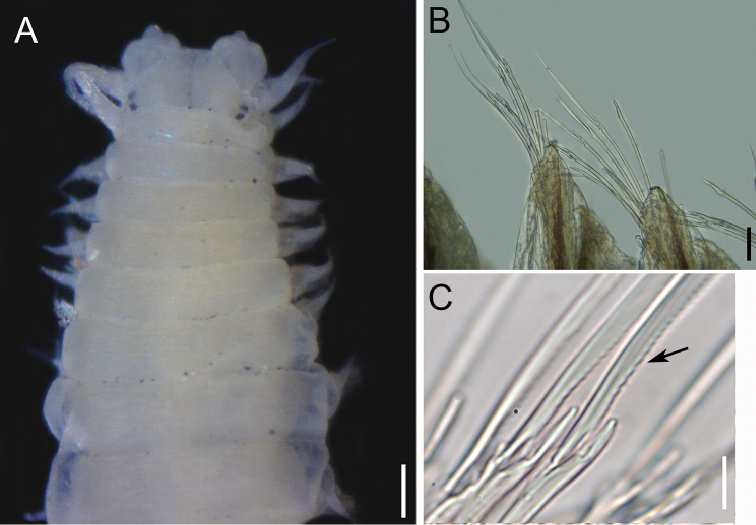
*Namalycastis geayi*, fixed specimen, NPM-Pol 882 and 884. **A** Anterior end, dorsal view **B** Parapodia sub-birramous, anterior region, dorsal view **C** Supra-acicular spiniger, arrow point to fine serrations proximally to the base of chaetae blades, parapodium 17, dorsal view of chaetae. Scale bars: 0.2 mm (**A**), 0.05 mm (**B**), 0.01 mm (**C**).

Based on specimen NPM-Pol 884. Incomplete specimen with 4.93 mm long, 1.1 mm wide and 18 chaetigers. Body widest mid-anteriorly, tapering gradually anteriorly and posteriorly (Fig. 6A). Dorsum and venter convex. Epidermal pigment absent. Prostomium trapezoidal, some individuals with lateral indentation on prostomium (Fig. 6A). Prostomium anterior end smooth. Antennae short and smooth, extending short of the anterior end of the prostomium, laterally inserted. Two pairs of eyes, arranged nearly longitudinally on prostomium. Four pairs of tentacular cirri with indistinct cirrophores and smooth cirrostyles. Posterodorsal pair extending posteriorly to third chaetiger. Pharynx smooth, lacking paragnaths or papillae. Parapodia sesquirramous (sub-birramous). Dorsal cirri short, similar in length throughout the body. Neuropodial acicular ligulae bilobed. Notochaeta as sesquigomph spinigers present from third chaetiger. Neurochaetae as heterogomph spinigers in all fascicles. Supra-acicular sesquigomph spinigers (postacicular) shaft with boss 1.9×–2.0× length of collar (Fig. 6B). Shaft of heterogomph chaeta with boss slightly prolonged. Sub-acicular spinigers in anterior region of body with blades moderately serrated (Fig. 6C). Chaeta pale. Aciculae dark brown.

######### Colour.

Specimens in alcohol yellow. No pigment visible throughout the body.

######### Remarks.

First species record for Brazil. The collected specimens of this study were not complete, but they present the same characters of *Namalycastis geayi* (Gravier, 1901) based on the anterior end (Fig. 6A). The identification of this species is supported by the presence of only heterogomph spinigers in sub- and supra-preacicular fascicle in the parapodia (Fig. 6B, C). In the original description, *N. geayi* has been recorded in freshwater environments, muddy river banks, and in coarse sediments (Gravier 1901). This study recorded *N. geayi* in mangroves and brackish water.

######## Namalycastis senegalensis

Taxon classificationAnimaliaPhyllodocidaNephtyidae

(Saint-Joseph, 1901)

Fig. 7

######### Type locality.

Marsassoun, Senegal (13°59'N, 16°43'W; estimated geolocation).

######### Material examined.

Caranguejos Island, 02°49'33.6"S, 44°28'51.1"W: one specimen, incomplete, 22 October 2010 (NPM-Pol 105).

**Figure 7.**
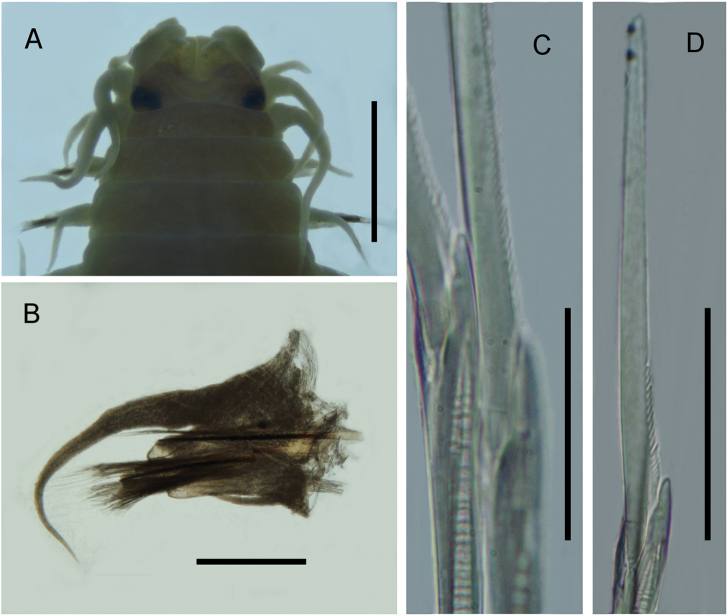
*Namalycastis senegalensis*, fixed specimen, NPM-Pol 105. **A** Anterior end, dorsal view **B** Parapodia of 10^th^ chaetiger, anterior view **C** Sub-acicular neurochaetae spiniger of 10^th^ chaetiger **D** Sub-acicular neurochaeta falciger of 10^th^ chaetiger. Scale bars: 2.3 mm (**A**), 0.2 mm (**B**), 0.02 mm (**C, D**).

######### Distribution.

Atlantic Ocean: Senegal, Nigeria, Congo, Suriname, Brazil (states of Pará and Maranhão, see Suppl. material 1).

######### Remarks.

First record for Maranhão. Complete specimens were not found in this study; however, the features of the anterior body are very similar to the re-description of Glasby (1999). The presence of thick cuticle covering the eyes, supra neuro acicular sesquigomph spinigers in the parapodia of chaetiger 10, with a 1.4 × length of collar or more boss, and distally smooth falciger blades supports the identification of the species. Previous Brazilian records include the Amazon coast, the estuarine beaches of Marajó Island in the mouth of the Amazon River (Glasby 1999), and the delta of the Amazon River (one specimen, ZHM PE405) (Glasby 1999). This species is known to live in brackish water and freshwater environments such as mangroves, creeks, and marshes (Glasby 1999).

###### Family Pilargidae Saint-Joseph, 1899

####### Genus *Sigambra* Müller, 1858

######## Sigambra bassi

Taxon classificationAnimaliaPhyllodocidaPilargidae

(Hartman, 1945)

Fig. 8A

######### Type locality.

Lemon Bay, Florida, USA (26°54'N, 82°20'W, estimated geolocation).

**Figure 8.**
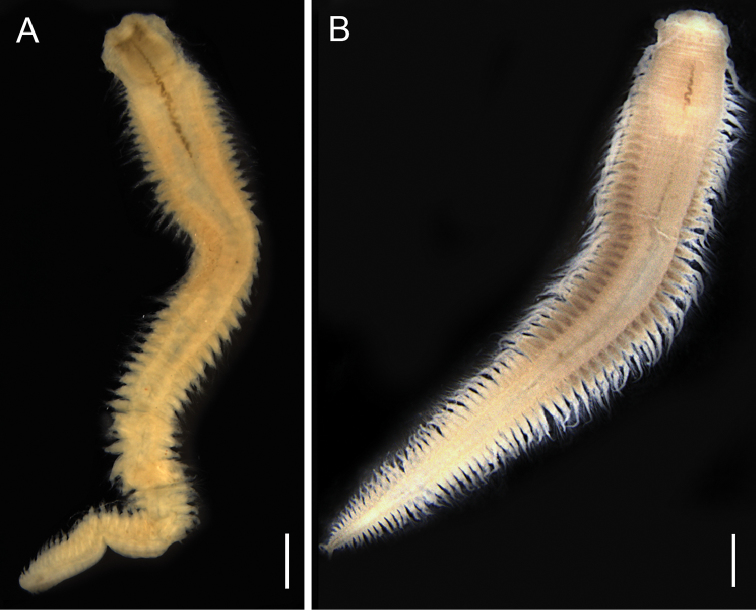
Pilargidae species. **A** *Sigambra bassi*, whole body, dorsal view **B** *Sigambra grubii*, whole body, dorsal view. Scale bars: 0.5 mm.

######### Material examined.

São Luís, 02°35'56"S, 44°21'11.8"W: one specimen, complete, 6 September 2011 (NPM-Pol 111).

######### Distribution.

Pacific Ocean: USA, Mexico, Chile. Atlantic Ocean: USA, Caribbean Sea, Brazil (state of Maranhão and São Paulo, see Suppl. material 1).

######### Remarks.

First record for Maranhão. The specimens examined in this study present a long medium antenna reaching up to setiger 5–12; a dorsal hook beginning in the posterior chaetigers supports the identification as *Sigambra bassi*. The records in the Caribbean and Brazil include estuaries and beaches (Gillet 1986, Amaral et al. 2003).

######## Sigambra grubii

Taxon classificationAnimaliaPhyllodocidaPilargidae

Müller in Grube, 1858

Fig. 8B

######### Type locality.

Florianópolis, Santa Catarina, Brazil (27°36'30”S, 48°26'30”W; original geolocation).

######### Material examined.

São Luís, 02°35'56"S, 44°21'11.8"W: one specimen, 18 August 2010 (NPM-Pol 110); one specimen, 27 January 2011 (NPM-Pol 887). Caranguejos Island, 02°49'33.6"S, 44°28'51.1"W: one specimen, 22 April 2010 (NPM-Pol 888). Complete and incomplete specimens.

######### Distribution.

Atlantic Ocean: USA, Caribbean Sea, Brazil (states of Pará, Maranhão, Sergipe, Rio de Janeiro, São Paulo, Santa Catarina and Rio Grande do Sul, see Suppl. material 1).

######### Remarks.

First species record for Maranhão. The presence of notopodial hooks distally curved appearing in setiger 20 and a medium antenna reaching the second chaetiger are characteristics that support the identification of the species based on the original description by Müller (1858) and re-description by Salazar-Vallejo (1990). In this study, the hooks appeared among the segments 6–29, in specimens shorter and with reduced number of chaetigers, the hooks appeared before the chaetiger 20. This type of variability in the hooks position related with the body size and number of chaetigers was also reported by Salazar-Vallejo (1990). No other morphological variation was found. This species is widely recorded in the coast of Brazil, mainly in estuarine environments, including mangroves and coastal lagoons as the type locality (Müller 1858). In the Caribbean, the species was recorded in a coastal lagoon (Liñero-Arana and Díaz-Díaz 2005).

###### Family Syllidae Grube, 1850

####### Genus *Exogone* Örsted, 1845

######## Subgenus Exogone (Exogone) Örsted, 1845

######### Exogone (Exogone) breviantennata

Taxon classificationAnimaliaPhyllodocidaSyllidae

Hartmann-Schröder, 1959

Fig. 9

########## Type locality.

Estero Jaltepeque, El Salvador (13°18'N, 88°52”W; estimated geolocation).

########## Material examined.

São Luís, 02°35'56"S, 44°21'11.8"W: one specimen, 18 August 2010 (NPM-Pol 889); three specimens, 27 January 2011 (NPM-Pol 890); one specimen, 29 March 2011 (NPM-Pol 112); one specimen, 6 September 2011 (NPM-Pol 891); five specimens, 18 December 2011 (NPM-Pol 892); all complete specimens.

**Figure 9.**
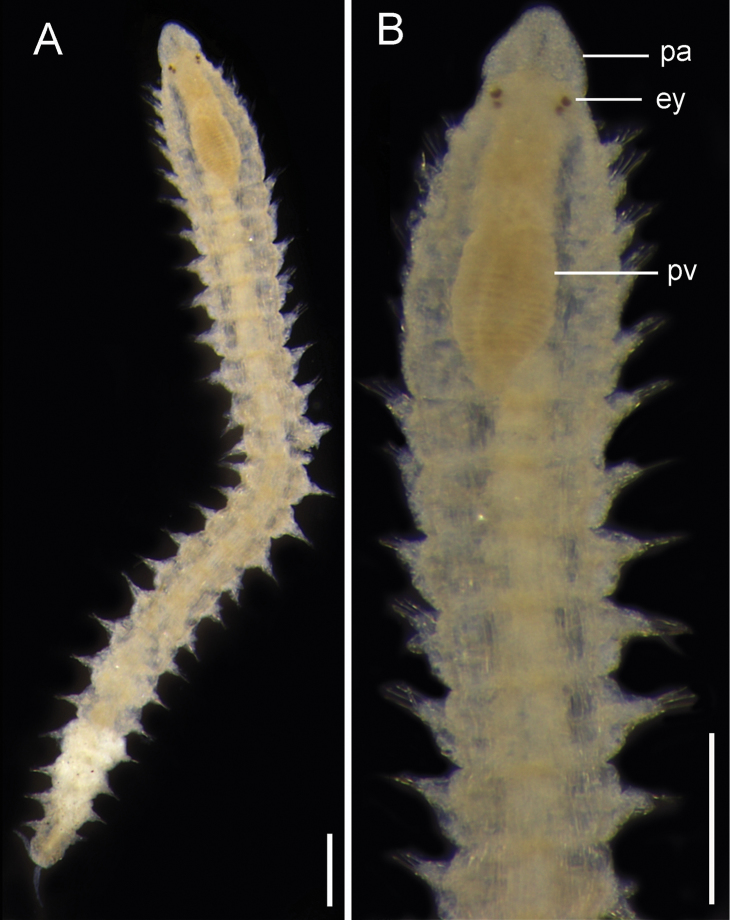
Exogone (Exogone) breviantennata. **A** Whole body, dorsal view **B** Anterior end, dorsal view. Abbreviations: pa, palps; ey, eye; pv, proventricle. Scale bars: 0.15 mm.

########## Distribution.

Pacific Ocean: Australia, Panama, Ecuador. Indian Ocean: Seychelles, Red Sea, Australia. Atlantic Ocean: Spain (Canary Islands), South Africa, Caribbean Sea, Brazil (states of Maranhão, Paraíba, Pernambuco, Espírito Santo, and São Paulo, see Suppl. material 1).

########## Remarks.

First species record for the Brazilian Amazon Coast. The features that confirm these specimens as Exogone (Exogone) breviantennata Hartmann-Schröder, 1959 are median and lateral antennae of similar size, compound spinigers and falcigers with bidentate blades (subdistal tooth larger than distal tooth) and falcigers in the anterior body with 3–4 relatively thick spines. This species is found worldwide in several habitats such as in seagrass in the intertidal zone, rocky shores, algae asSEMblages, soft bottoms (San Martín and Bone 2001, Paresque et al. 2014), and others. The type material of E. (E.) breviantennata is from a mangrove (Hartmann-Schröder 1959) as in the present study. However, this species has been recorded in several environments and it presents a circumtropical distribution (Núñez et al. 1992).

##### Subclass Sedentaria

###### Order Terebellida

####### Family Ampharetidae Malmgren, 1866

######## Genus *Isolda* Müller, 1858

######### Isolda pulchella

Taxon classificationAnimaliaTerebellidaAmpharetidae

Müller, 1858

Fig. 10A–C

########## Type locality.

Florianópolis, Santa Catarina, Brazil (27°36'S, 48°27'W; estimated geolocation).

########## Material examined.

São Luís, 02°35'56" S, 44°21'11.8"W: seven specimens, 6 September 2011 (NPM-Pol 849); two specimens, 18 December 2012 (NPM-Pol 067); 14 specimens, 18 December 2012 (NPM-Pol 848). Caranguejos Island, 02°49'33.6"S, 44°28'51.1"W: one specimen, 17 December 2012 (NPM-Pol 850). Complete and incomplete specimens.

**Figure 10.**
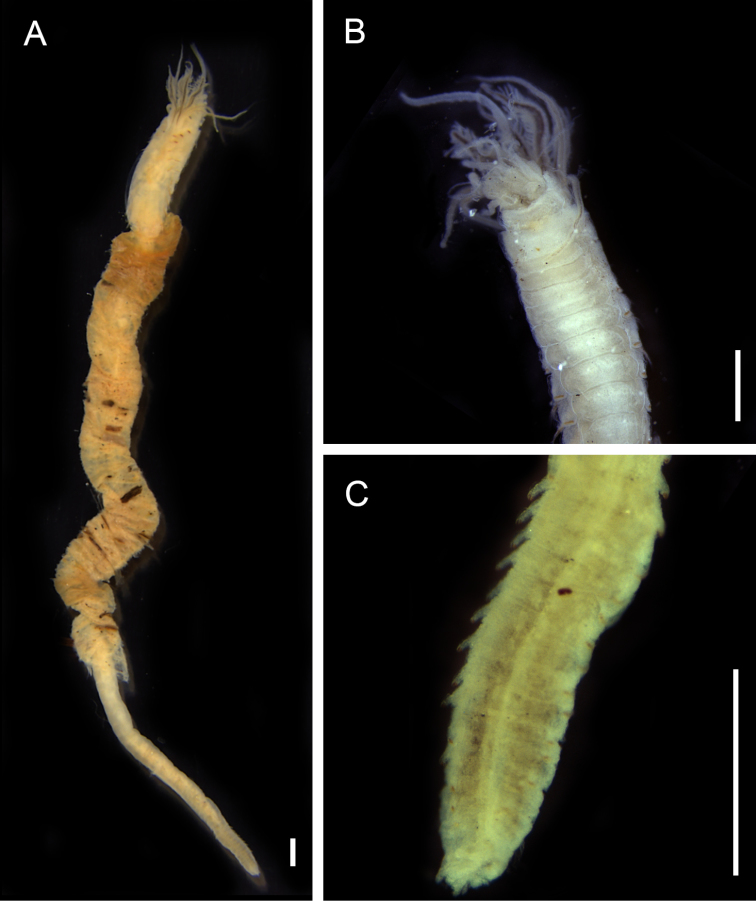
*Isolda pulchella*. **A** Whole body, dorsolateral view **B** Anterior end, dorsal view **C** Pygidium, ventral view. Scale bars: 0.5 mm.

########## Distribution.

Pacific Ocean: Australia, USA. Indian Ocean: Red Sea, Australia. Atlantic Ocean: Portugal, Mediterranean Sea, South Africa, USA, Mexico, Caribbean Sea, Brazil (states of Pará, Maranhão, Sergipe, Rio de Janeiro, São Paulo, Paraná and Santa Catarina, see Suppl. material 1).

########## Remarks.

The presence of two groups of four branchiae, post-branchial notopodium with sharply curved hooks; twelve or thirteen thoracic segments with pectinate uncini with four to seven teeth support the identification of these specimens as *Isolda pulchella* Müller, 1858. This species was described in south Brazil and is found along the coast inhabiting estuarine environments, including mangroves and coastal lagoons. The specimens described by Díaz-Díaz and Liñero-Arana (2012) for Caribbean Sea are also similar to the specimens in this study and are recorded in estuaries.

###### Infraclass Scolecida

####### Family Capitellidae Grube, 1862

######## Genus *Capitella* Blainville, 1828

######### Capitella capitata

Taxon classificationAnimaliaCapitellidaCapitellidae

(Fabricius, 1780), complex

Fig. 11A, B

########## Type locality.

Uummannaq, West Greenland (71°6.5'N, 51°17'W; original geolocation).

########## Material examined.

São Luís, 02°35'56"S, 44°21'11.8"W: six specimens, 29 March 2011 (NPM-Pol 069); one specimen, 23 September 2012 (NPM-Pol 102); 17 specimens, 18 December 2011 (NPM-Pol 851). Caranguejos Island, 02°49'33.6"S, 44°28'51.1"W: five specimens, 17 October 2010 (NPM-Pol 852); two specimens, 26 January 2011 (NPM-Pol 853); two specimens, 17 December 2011 (NPM-Pol 854). Complete and incomplete specimens.

**Figure 11.**
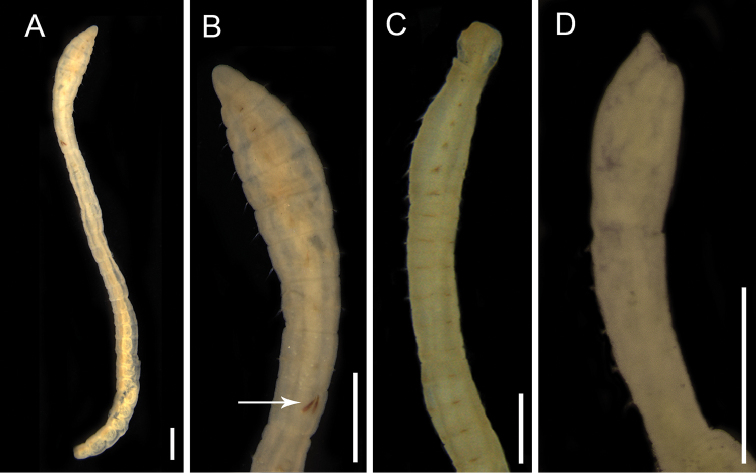
Capitellidae species. **A** *Capitella capitata* complex, whole body, lateral view and **B** anterior view, arrow point genital spines in 9^th^ chaetiger **C** *Heteromastus filiformis*, thoracic region, lateral view **D** *Mediomastus californiensis*, thoracic region, lateral view. Scale bars: 0.5 mm.

########## Distribution.

Arctic Ocean: Greenland. Pacific Ocean: China, Japan, Australia, USA, Mexico, Costa Rica. Indian Ocean: Red Sea. Atlantic Ocean: North Sea, Ireland, UK, Germany, Netherlands, France, Spain, Mediterranean Sea, Ukraine, South Africa, USA, Mexico, Caribbean Sea, Brazil (states of Pará, Maranhão, Ceará, Paraíba, Rio de Janeiro, São Paulo, Paraná, Santa Catarina and Rio Grande do Sul, see Suppl. material 1).

########## Remarks.

First record for Maranhão. The specimens examined in this study are similar to the neotype description of *Capitella capitata* by Blake (2009). *C. capitata* was considered as a globally distributed species, but allozyme analyses have demonstrated that this species is composed of at least six sibling species (Grassle and Grassle 1976). Additionally, Blake (2009) indicates that *C. capitata* may be only distributed in Arctic regions, suggesting that the numerous records from lower latitudes are not this species. Nevertheless, the specimens from warmer waters such as in the Caribbean (Amoureux 1985) and Brazil (Pardo et al. 2010) are also similar to the neotype descriptions by Blake (2009). Recently, four new species of *Capitella* were described from the *Capitella capitata* complex (Silva et al. 2017). Studies including molecular data must to be conducted on these animals from Maranhão, and the specimens should be re-examined.

######## Genus *Heteromastus* Eisig, 1887

######### Heteromastus filiformis

Taxon classificationAnimaliaCapitellidaCapitellidae

(Claparède, 1864)

Fig. 11C

########## Type locality.

Port-Vendres, France (42°30'N, 3°07'E; estimated geolocation).

########## Material examined.

São Luís, 02°35'56"S, 44°21'11.8"W: one specimen, incomplete, 6 September 2011 (NPM-Pol 070); two specimens, 27 January 2011 (NPM-Pol 852); four specimens, 18 December 2011 (NPM-Pol 856); one specimen, 18 March 2012 (NPM-Pol 857); complete and incomplete specimens.

########## Distribution.

Pacific Ocean: New Zealand, USA, Costa Rica. Indian Ocean: Red Sea, Mozambique. Atlantic Ocean: Ireland, UK, Belgium, France, Mediterranean Sea, South Africa, USA, Mexico, Caribbean Sea, Brazil (states of Pará, Maranhão, Bahia, Rio de Janeiro, São Paulo, see Suppl. material 1).

########## Remarks.

*Heteromastus filiformis* from São Marcos Bay share the same characters of the specimens described by Day (1967) and Dean (2001) such as thoracic region with 12 segments, the first achaetous; thoracic hooks with long hood and about six denticles above the main tooth; abdominal hooks narrow and three to four denticles above the main tooth, gills in subsequent medial segments. The specimens of *H. filiformis* examined in this study are very similar to *H. similis* Southern, 1921. One of the main differences between those species is the presence of gills processes and the shape of neuropodial hooks in *H. filiformis*. According to Hartman (1947), *Heteromastus similis* is considered an inhabitant of freshwater areas and *H. filiformis* is typical of marine environments. Both species have distribution in estuarine environments such as mangroves from Brazil (Silva et al. 2011). In the Caribbean Sea, the records are also in estuarine areas and especially in the muddy intertidal areas of the Caribbean Sea (Gobin 1990). Both species seems to be distributed worldwide, independent of environmental salinity, but descriptions based on fewer characters can be related to several records around the world.

######## Genus *Mediomastus* Hartman, 1944

######### Mediomastus californiensis

Taxon classificationAnimaliaCapitellidaCapitellidae

Hartman, 1944

Fig. 11D

########## Type locality.

Tomales Bay, California (38°18'N, 122°56'W; estimated geolocation).

########## Material examined.

São Luís, 02°35'56"S, 44°21'11.8"W: two specimens, 21 October 2010 (NPM-Pol 73); three specimens, 18 August 2010 (NPM-Pol 858); one specimen, 18 March 2012 (NPM-Pol 859). Caranguejos Island, 02°49'33.6"S, 44°28'51.1"W: one specimen, 22 April 2010 (NPM-Pol 860), three specimens, 26 January 2011 (NPM-Pol 861); three specimens, 28 March 2011 (NPM-Pol 862); eight specimens, 28 March 2011 (NPM-Pol 863); one specimen, 17 December 2011 (NPM-Pol 864); four specimens, 2 July 2012 (NPM-Pol 865). Complete and incomplete specimens.

########## Distribution.

Pacific Ocean: Australia, USA, Mexico. Atlantic Ocean: Caribbean Sea, Brazil (states of Pará, Maranhão, Rio de Janeiro, São Paulo, Paraná and Santa Catarina, see Suppl. material 1).

########## Remarks.

First record for Maranhão. The specimens examined in this study have triangular prostomium with cylindrical palpodium, in dorsal view; peristomium devoid of setae with a pair of ocelli; 10 chaetigers in thoracic region; only capillaries in chaetigers 1–4; abdominal chaetigers only with hooded hooks defining them as *Mediomastus californiensis* (Hartman, 1944). In the present study, we found specimens exceeding 100 segments as observed by Warren et al. (1994). Although *M. californiensis* has been recorded in the Pacific (USA) and Atlantic Ocean (Canada and the USA), Warren et al. (1994) examined specimens from both oceans and did not observe differences among them. This species has been recorded in muddy bottoms of estuarine environments in the Brazilian Amazon Coast (Rosa-Filho et al. 2006) and in the Caribbean Sea (Gobin 1990).

####### Family Paraonidae Cerruti, 1909

######## Genus *Paraonis* Cerruti, 1909

######### Paraonis amazonica sp. n.

Taxon classificationAnimaliaCapitellidaParaonidae

http://zoobank.org/D7449E5D-1126-4135-A4B2-DB76AE4CFCCE

Figs 12 , 13 , 14

########## Type locality.

Brazil, Maranhão: São Luís, 02°35'56"S, 44°21'11.8"W, mangrove, 21 October 2010, R.P. Ribeiro.

########## Material examined.

Holotype: São Luís, 02°35'56"S, 44°21'11.8"W, one specimen, complete, 21 October 2010 (NPM-Pol 906). Paratypes: São Luís, 02°35'56"S, 44°21'11.8"W, one specimen, incomplete, 18 August 2010 (NPM-Pol 907); 80 specimens, all incomplete, 27 January 2011 (NPM-Pol 908); two specimens, both complete, 21 October 2010 (NPM-Pol 929); two specimens, both incomplete, 18 March 2012 (MNCN 16.01/17766). Caranguejos Island, 02°49'33.6"S, 44°28'51.1"W, three specimens, all incomplete, 26 January 2011 (NPM-Pol 930); 11 specimens, all incomplete, 28 March 2011 (MNCN 16.01/17765).

########## Distribution.

Only known from the type locality.

########## Diagnosis.

Rounded prostomium, clearly wider than longer. Dorsal brownish pigmentation reaching the beginning of the prostomium. Rounded to foliaceous branchiae (4–8 pairs), from the fourth segment. Neurochaetae of two types: acicular chaeta with lateral spine beginning in pre-branchial segments, and hook-shaped chaeta with terminal spines in post-branchial segments.

########## Description.

**Figure 12.**
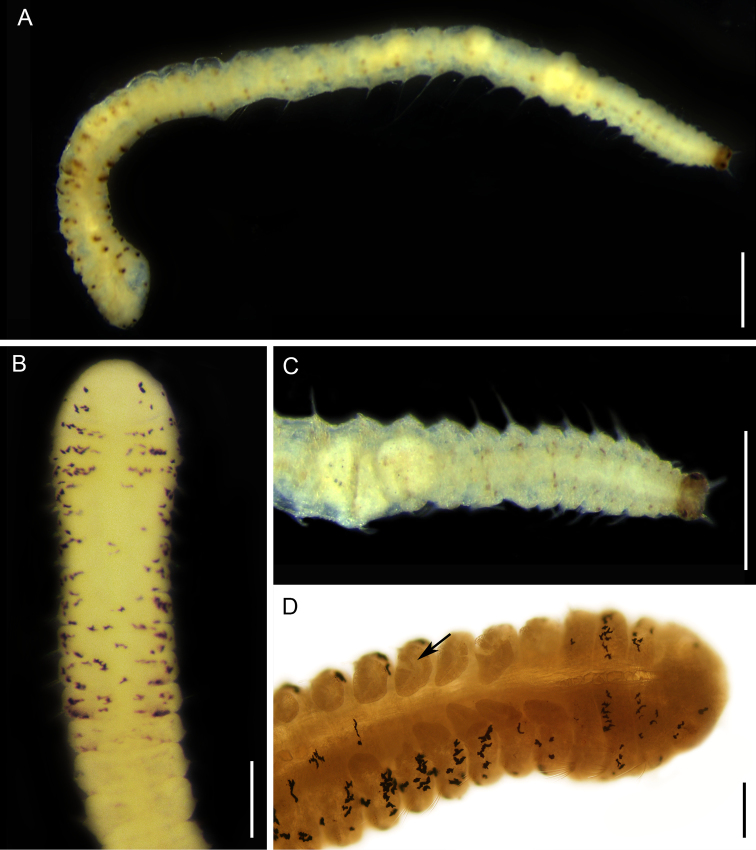
*Paraonis amazonica* sp. n., fixed specimen, NPM-Pol 906. **A** Whole body, lateral view **B** Anterior end, dorsal view **C** Posterior end, dorsal view **D** Anterior end, the arrow indicates foliaceous branchiae, dorsolateral view. Scale bars: 0.5 mm (**A**), 0.1 mm (**B**) 0.25 mm (**C, D**).

**Figure 13.**
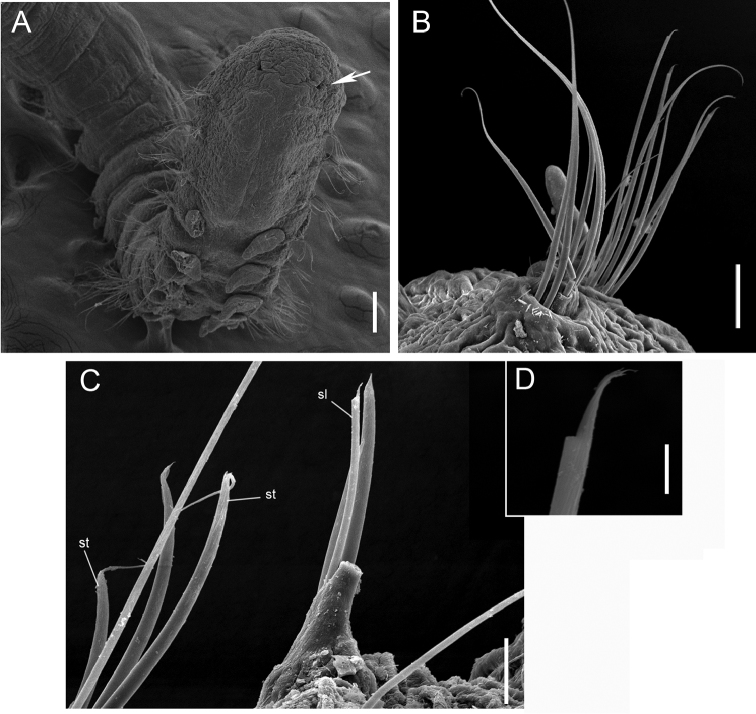
*Paraonis amazonica* sp. n., SEM. **A** Anterior end, arrow point to the nuchal organ, dorsal view **B** Anterior parapodium of chaetiger 3, arrow point to the acicular lateral spine, dorsal view **C** Parapodium of setiger 35, acicular chaeta with a lateral spine (**sl**) hook-shaped chaeta with a terminal spine (**st**) **D** Acicular chaeta with a lateral spine enlarged. Scale bars: 0.1 mm (**A**), 20 µm (**B**), 10 µm (**C**), 1 µm (**D**).

**Figure 14.**
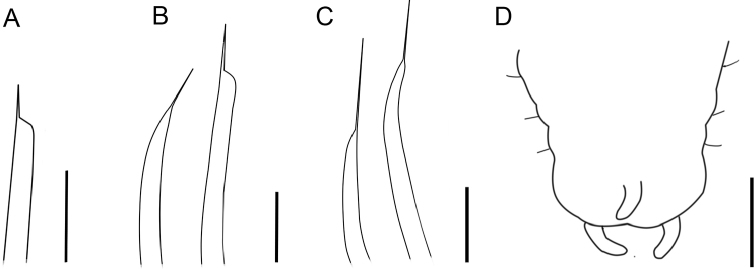
*Paraonis amazonica* sp. n., chaetae. **A** Acicular chaetae with a lateral spine of anterior setiger 3 **B** Posterior hook-shaped chaeta with terminal spine and acicular chaetae with a lateral spine (left) in setiger 30 **C** Hook-shaped chaeta with a terminal spine in the last setiger, 36 **D** Pygidium with three anal cirri, ventral view. Scale bars 50 µm (**A–C**), 0.25 mm (**D**).

Complete holotype, 2.68 mm long, 0.17 mm wide (chaetiger 8), and 46 chaetigers. Three complete paratypes with 2.43–2.94 mm long, 0.18–0.20 mm wide and 36–54 chaetigers. Incomplete paratypes up to 4.607 mm long, 0.283 mm wide, and 16–61 chaetigers. Fixed individuals with brown pigmentation that reaches the distal end of the prostomium and extends along the body. Anteriorly flattened body, wider than longer, cylindrical from the 8^th^ chaetiger and in all middle body region (Fig. 12A, B). Branchial region dorsoventrally flattened. Rounded prostomium, wider than longer (Fig. 12B, D). Absence of antenna, palpode, ciliated bands and eyes in the prostomium (Fig. 12A, B). The anterior segments are short, wider than longer. Long and biannulate segments in the post-branchial region. One pair of nuchal organs located on the posterior edge of the prostomium (Fig. 13A). Notopodial post-chaetal lobes absent in the pre-branchial region, the first notopodial post-chaetal lobe appear in the fifth branchial chaetiger. Notopodial post-chaetal cirrifom lobes, longer from the middle and posterior regions. Branchiae from chaetiger 4, rounded to foliaceous, flat, short, 4–8 pairs, first and last pairs are shorter (Fig. 12D). Notopodial capillary chaetae throughout the body. Curved capillary chaetae in the neuropodium and notopodium of the pre-branchial and branchial segments (Fig. 13B). Capillary neurochaetae progressively thinner, longer, and straight in the post-branchial segments. Capillary notochaetae of the posterior segments thicker than those anterior and median segments. Pre-branchial and branchial segments with 3–5 chaetae capillaries in the notopodium and 2–5 in the neuropodium. Post-branchial segments with 1–2 chaetae capillary in the notopodium, absent in the neuropodium. First acicular neuropodial chaetae with a lateral spine in chaetiger 2–8, and 2–3 chaetae in the branchial segments (Figs 13B, 14A). Neuropodium in the post-branchial middle segments and posterior end segments with one acicular chaeta with a lateral spine (Figs 13B, C, 14A–C). Hook-shaped neurochaetae with terminal spine beginning in post-branchial chaetigers, 1–2 chaetae. Neuropodium in the post-branchial middle chaetigers with 2–4 hook-shaped chaetae with a terminal spine. Neuropodium in posterior chaetigers with two hook-shaped chaetae with a terminal spine (Figs 13B, C, 14B, C). Pygidium rounded with two anal lobes and three anal cirri: two dorsolateral and one medium-ventral (Figs 12C, 14D).

########## Colour.

Specimens in alcohol show brownish pigment spots all over the body, two pairs of reddish brown lateral spots in the pygidial lobes of some specimens.

########## Etymology.

Named after the Amazon Coast, region where type locality is located.

########## Remarks.

*Paraonis amazonica* sp. n. differs from all other species by the presence of acicular and hook-shaped modified neurochaetae. Currently, there are five valid species named in the genus *Paraonis*: *Paraonis fulgens* (Levinsen, 1884); *Paraonis paucibranchiata* Cerruti, 1909; *Paraonis pycnobranchiata* Fauchald, 1972; *Paraonis pygoenigmatica* Jones, 1968; and *Paraonis strelzovi* Hartmann-Schröder, 1980 (see Table 1). Several species first described as *Paraonis* were established as a synonymy of *Aricidea* (López 2008), *Levinsenia* (Gaston 1984), *Paradoneis* (Mackie 1991), and *Paraonides* (Parapar et al. 2012). *Paraonis tenera* Grube, 1873 is a species considered *nomen oblitum* by Strelzov (1973) because its description was inaccurate, being based on a single specimen and probably referring to a species of *Aricidea*.

**Table 1.** Key features of *Paraonis* based on original descriptions and redescriptions. NI: no information.

Features	Species					
	*P. amazonica* sp. n.	*P. fulgens* (Levinsen, 1884)	*P. paucibranchiata* Cerruti, 1909	*P. pycnobranchiata* Fauchald, 1972	*P. pygoenigmatica* Jones, 1968	*P. strelzovi* Hartmann-Schröder, 1980
Eyes	absent	present	present	present	present	absent
Prostomial ciliated bands	absent	present	absent	absent	present	NI
First chaetiger with branchiae	4	4	4	6	6	4
Number of branchiae pairs	4–8	16–25	4	20	15–19	4
Branchiae shape	foliaceous to rounded	foliaceous to oval	cylindrical	thick and distally blunt	Lanceolate	large, smooth and ciliated
Prostomium	rounded	conical	ovoid	rounded pentagonal	Conical	conical
Number of chaetigers	36–54	110–120	at least 20	at least 48	62–81	> 27
Notochaetae	capillary	capillary	capillary	capillary	capillary or limbate	capillary and capillary fringed
Pre-branchial and branchial neurochaetae	capillary and acicular with lateral spine	capillary and hook-shaped with fringe	capillary and hook-shaped	capillary	capillary or limbate	capillary and capillary fringed and hooded spine
Post-branchial neurochaeta	acicular with lateral spine and hook-shaped with terminal spine	hook-shaped with fringe	hook-shaped	curved and pilose without aristae	capillary or limbate and modified	hooded spine
Number of anal cirri	3	3	3	NI	3 to 8	3
Habitat	estuarine, intertidal	marine, intertidal	marine	deep sea	marine, subtidal	estuarine, subtidal
Bottom	muddy	sand bottom	NI	NI	sand bottom	NI
Type locality	Amazon Coast, Brazil	Denmark	Mediterranean Sea	Gulf of California, USA	Cape Cod Bay, USA	Australia

Among the five valid species of *Paraonis*, *P. fulgens*, *P. paucibranchiata*, and *P. strelzovi* also have the first pair of branchiae in the fourth chaetiger as seen in *P. amazonica* sp. n. However, *P. fulgens* has more than 25 pairs of branchiae and the first post-chaetal lobe starts in the third chaetiger, whereas *P. amazonica* sp. n. has 4–8 pairs of branchiae and first post-chaetal lobe in the 9^th^ chaetiger. In addition, *P. fulgens* (about 120 chaetigers in total) seems to be longer than *P. amazonica* sp. n. (up to 54 chaetigers in complete individuals). However, longer animals could be found, since incomplete individuals of *P. amazonica* sp. n. showed up to 61 chaetigers. Only four pairs of branchiae are described in *P. paucibranchiata* and *P. strelzovi* whereas *P. amazonica* sp. n. has 4–8 pairs of branchiae. Moreover, *P. paucibranchiata* differs from *P. amazonica* sp. n. by the presence of eyes and longer and straighter branchiae. The other two species mainly differ on the first chaetiger with branchiae and post-chaetal lobe. *Paraonis pygoenigmatica* has approximately 20 pairs of branchiae that begin in the sixth chaetiger, joined to the first dorsal lobes. In *P. pycnobranchiata*, the branchiae (about 19) are present from chaetiger 6–25. *P. amazonica* sp. n. and *P. pycnobranchiata* have the same pigmentation pattern consisting in small pigment spots scattered along the body.

Species of *Paraonis* are usually reported in marine, inshore and continental shelf environments (Glasby and Wilson 2003). There are some exceptions, such as *P. fulgens*, recorded in the intertidal zone from Caribbean Sea (Helguera et al. 2011), *P. strelzovi* in mangroves from Australia (Hartmann-Schröder 1980), and P. py*goenigmatica* recorded in estuarine areas from Brazil (Barros et al. 2001). *Paraonis amazonica* sp. n. is the first record of a *Paraonis* species found in muddy bottoms in mangrove vegetated areas.

## Conclusion

In total, 14 species belonging to eight families and eleven genera were identified in São Marcos Bay, Maranhão, Brazilian Amazon Coast. Two of them were first recorded to Brazilian Coast (*N. fauveli*, *N. geayi*) and one new species was described (*P. amazonica* sp. n.). Two other species are new records for the Brazilian Amazon Coast (E. (E.) breviantennata and *S. bassi*), and five species are new records for the Maranhão Coast (*C. capitata* complex, *M. californiensis*, *N. senegalensis*, *N. simoni*, and *S. grubii*).

This study expands the occurrence of *N. geayi* to the Brazilian Amazon Coast (in estuarine muddy sediments) because the type specimens of *N. geayi* were collected in freshwater and muddy bottoms in the Ouanary Stream in French Guiana (Gravier 1901). In addition, a new species of *Paraonis* is described in Amazon mangroves, although Paraonidae is a family commonly found and highly diversified in deep-sea environments (Aguirrezabalaga and Gil 2009). We encourage further studies on this genus because many species need improved descriptions, given that some features lack information in the original descriptions.

In summary, this checklist increases the number of recorded species in the Brazilian Amazon Coast. Further studies targeting sampling beyond mangroves and soft bottoms, including deep sea, seagrasses, and algal mats, can lead to the discovery of higher diversity of annelids in the Brazilian Amazon Coast. We assume that other new species can be found in this region or described from the worldwide species reported here, since they probably correspond to species complexes.

## Supplementary Material

XML Treatment for Arabella (Arabella) iricolor

XML Treatment for Nephtys simoni

XML Treatment for Alitta succinea

XML Treatment for Namalycastis fauveli

XML Treatment for Namalycastis geayi

XML Treatment for Namalycastis senegalensis

XML Treatment for Sigambra bassi

XML Treatment for Sigambra grubii

XML Treatment for Exogone (Exogone) breviantennata

XML Treatment for Isolda pulchella

XML Treatment for Capitella capitata

XML Treatment for Heteromastus filiformis

XML Treatment for Mediomastus californiensis

XML Treatment for Paraonis amazonica

## Supplementary materials

Supplementary material 1 Brazilian records of the species identified from Amazon coast, Maranhão Data type: species data Explanation note: Information on deposit number, record, state, coordinates, habitat, substrate and depth and references of the records are provided. This is an open access article distributed under the terms of the Creative Commons Attribution License (CC BY 4.0), which permits unrestricted use, distribution, and reproduction in any medium, provided the original author and source are credited.Rannyele Passos Ribeiro, Paulo Ricardo Alves, Zafira da Silva de Almeida, Christine Ruta
